# Supplementary figures and images for: Community-Based Workshops to Involve Rural Communities in Wildlife Management Case Study: Bighorn Sheep in Baja California, Mexico
Source: Animals (Basel). 2023 Oct 11;13(20):3171. doi: 10.3390/ani13203171 (PMC10603732; doi:10.3390/ani13203171)

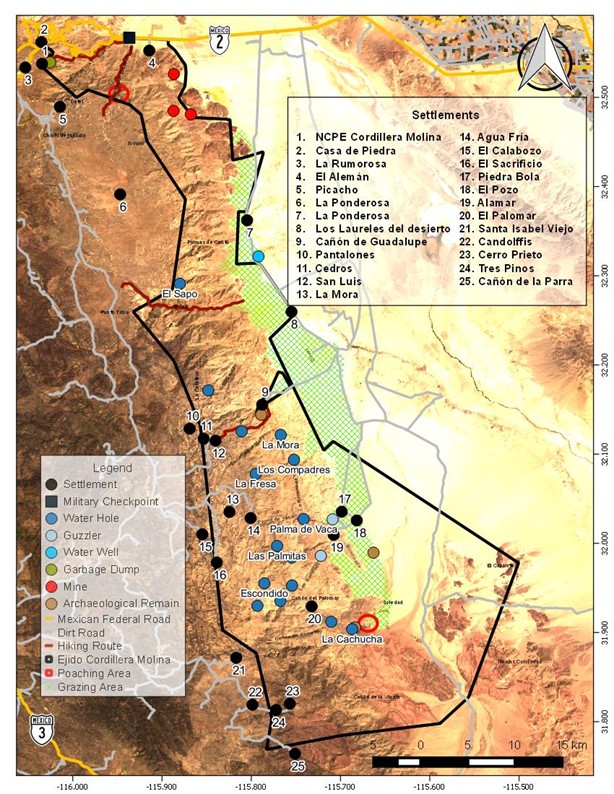

Supplement: Supplementary file 1 [file animals-13-03171-s001.zip › Figure S1.jpg]

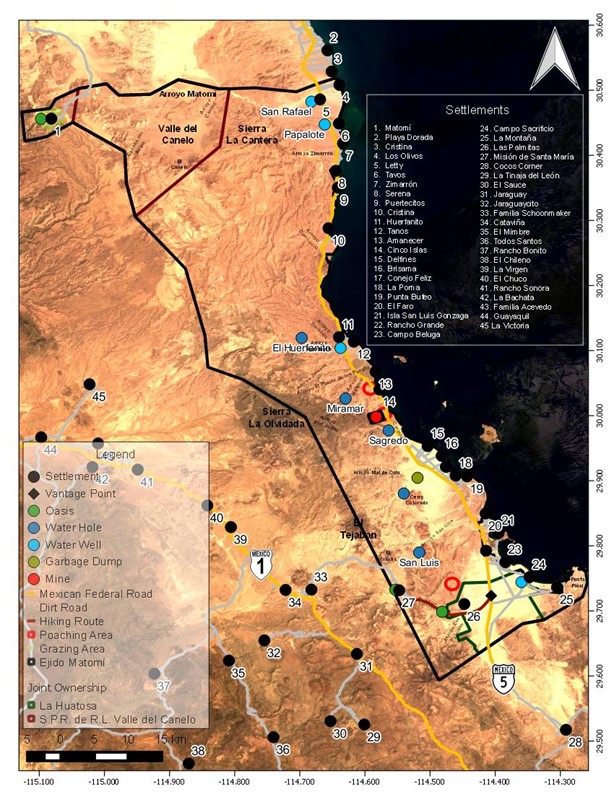

Supplement: Supplementary file 1 [file animals-13-03171-s001.zip › Figure S2.jpg]

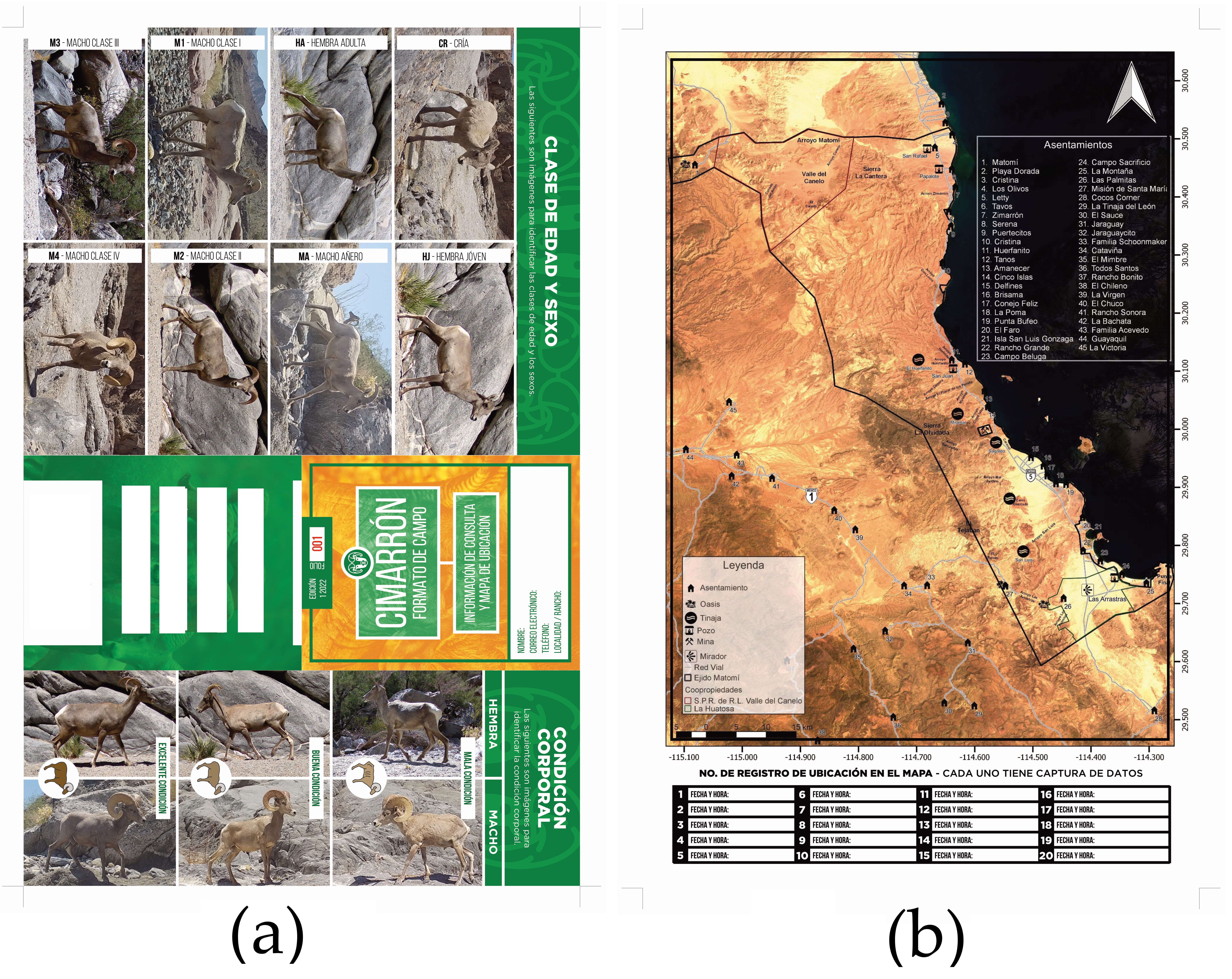

Supplement: Supplementary file 1 [file animals-13-03171-s001.zip › Figure S4.jpg]
